# Insertion sites of the muscles attached to the clavicle: a cadaveric study of the clavicle

**DOI:** 10.1186/s12891-023-06266-4

**Published:** 2023-03-03

**Authors:** Hiroyuki Imazato, Nobuyasu Takahashi, Akira Sawaguchi, Yusuke Hirakawa, Yoichiro Yamaguchi, Masaru Hiyoshi, Takuya Tajima, Etsuo Chosa

**Affiliations:** 1 Division of Orthopaedic Surgery, Department of Medicine of Sensory and Motor Organs, Faculty of Medicine, University of Miyazaki, Miyazaki, Japan; 2 Department of Anatomy, Ultrastructural Cell Biology, Faculty of Medicine, University of Miyazaki, 5200 Kihara, Kiyotake, Miyazaki Japan

**Keywords:** Anatomical structures, Anterior plate, Clavicle, Muscle insertion sites, Muscle attachments, Quantitative 3D analysis

## Abstract

**Background:**

Clavicle fractures are common injuries, especially in young, active individuals. Operative treatment is recommended for completely displaced clavicle shaft fractures, and plate fixation is stronger than the use of intramedullary nails. Few studies have reported on iatrogenic injuries to the muscle attached to the clavicle during fracture surgery. The aim of this study was to clarify the area of the insertion sites of muscles attached to the clavicle in Japanese cadavers using gross anatomy and three-dimensional (3D) analysis. We also aimed to compare the effects of anterior plate templating and superior plate templating on clavicle shaft fractures using 3D images.

**Methods:**

Thirty-eight clavicles from Japanese cadavers were analyzed. We removed all clavicles to identify the insertion sites and measured the size of the insertion area of each muscle. Three-dimensional templating was performed on both the superior and anterior plates of the clavicle using data obtained from computed tomography. The areas covered by these plates on the muscles attached to the clavicle were compared. Histological examination was performed on four randomly selected specimens.

**Results:**

The sternocleidomastoid muscle was attached proximally and superiorly; the trapezius muscle was attached posteriorly and partly superiorly; and the pectoralis major muscle and deltoid muscles were attached anteriorly and partially superiorly. The non-attachment area was located mainly in the posterosuperior part of the clavicle. It was difficult to distinguish the borders of the periosteum and pectoralis major muscles. The anterior plate covered a significantly broader area (mean 6.94 ± 1.36 cm^2^) of the muscles attached to the clavicle than did the superior plate (mean 4.11 ± 1.52 cm^2^) (p < 0.0001). On microscopy, these muscles were inserted directly into the periosteum.

**Conclusion:**

Most of the pectoralis major and deltoid muscles were attached anteriorly. The non-attachment area was located mainly from the superior to posterior part of the clavicle midshaft. Both macroscopically and microscopically, the boundaries between the periosteum and these muscles were difficult to demarcate. The anterior plate covered a significantly broader area of the muscles attached to the clavicle than that by the superior plate.

## Introduction

### Background

Clavicle fractures are common injuries, especially in young, active individuals, and account for 2.6% of all fractures [7]. Approximately 80% of clavicle fractures occur in the midshaft of the bone [7]. They are likely to occur in male individuals under 30 years of age, typically as a result of collision sports, such as football, hockey, rugby, high-speed falls from motorcycles, and traffic accidents [4, 18, 21].

Operative treatment is recommended for completely displaced clavicle shaft fractures [2, 7]. A meta-analysis of randomized controlled trials showed that operative treatment is a good treatment option for patients who require rapid functional recovery and those with risk factors for nonunion, such as comminution and displacement [25]. Intramedullary nails and anterior and superior plates are used for open reduction and internal fixation of clavicle fractures [1, 5, 15]. Generally, both implants are used for displaced clavicle fractures; intramedullary nails are used when there is a need for decreasing complications [7, 15, 17], while plates are used for stronger fixation [13]. Anatomically, six types of muscles are identified around the clavicle: the platysma muscle stops in front of the clavicle and wraps around the clavicle, and the sternocleidomastoid, pectoralis major, deltoid, trapezius, and subclavius muscle are attached to the clavicle [11, 14]. The soft tissues surrounding the clavicle are injured to some extent during operative procedures, such as detachment and dissection of the muscles. Iatrogenic injuries to the supraclavicular nerve, subclavian artery, and subclavian vein during clavicle fracture surgery have been reported [6, 12, 24]. Nevertheless, few studies on muscle injury have been conducted to date.

The purpose of this study was to clarify and evaluate the area of insertion sites of muscles attached to the clavicle in Japanese cadavers using gross anatomy and three-dimensional (3D) analysis. Furthermore, we aimed to compare the effect of anterior plate templating on clavicle shaft fractures with that of the superior plate templating using 3D images.

## Materials and methods

### Sample preparation for cadaveric anatomy

This study was approved by the ethics board of the Faculty of Medicine, University of Miyazaki (approval number: O-1049) and written informed consent was obtained from all cadavers and their families when they had registered as a donor in University of Miyazaki. Thirty-eight human clavicles of Japanese cadavers (15 male and 23 female cadavers; 23 right and 15 left; mean age at death, 83.9 years) donated to the Department of Anatomy were used in this study.

All cadavers were fixed with 10% formalin and preserved in 70% ethanol. The sternocleidomastoid, trapezius, pectoralis major, and deltoid muscles were cut with a 10 mm margin from their insertion on the clavicle. After cutting the muscles, the acromioclavicular joint capsule, sternoclavicular joint capsule, and coracoclavicular ligament were sectioned. We simply sectioned the subclavius muscle, as it does not directly affect the approach for osteosynthesis of clavicle fractures. All connective tissues overlying the muscles were removed to accurately identify the insertion site.

### Measurement and evaluation of insertion sites of the muscles attached to the clavicle

**Fig. 1:**
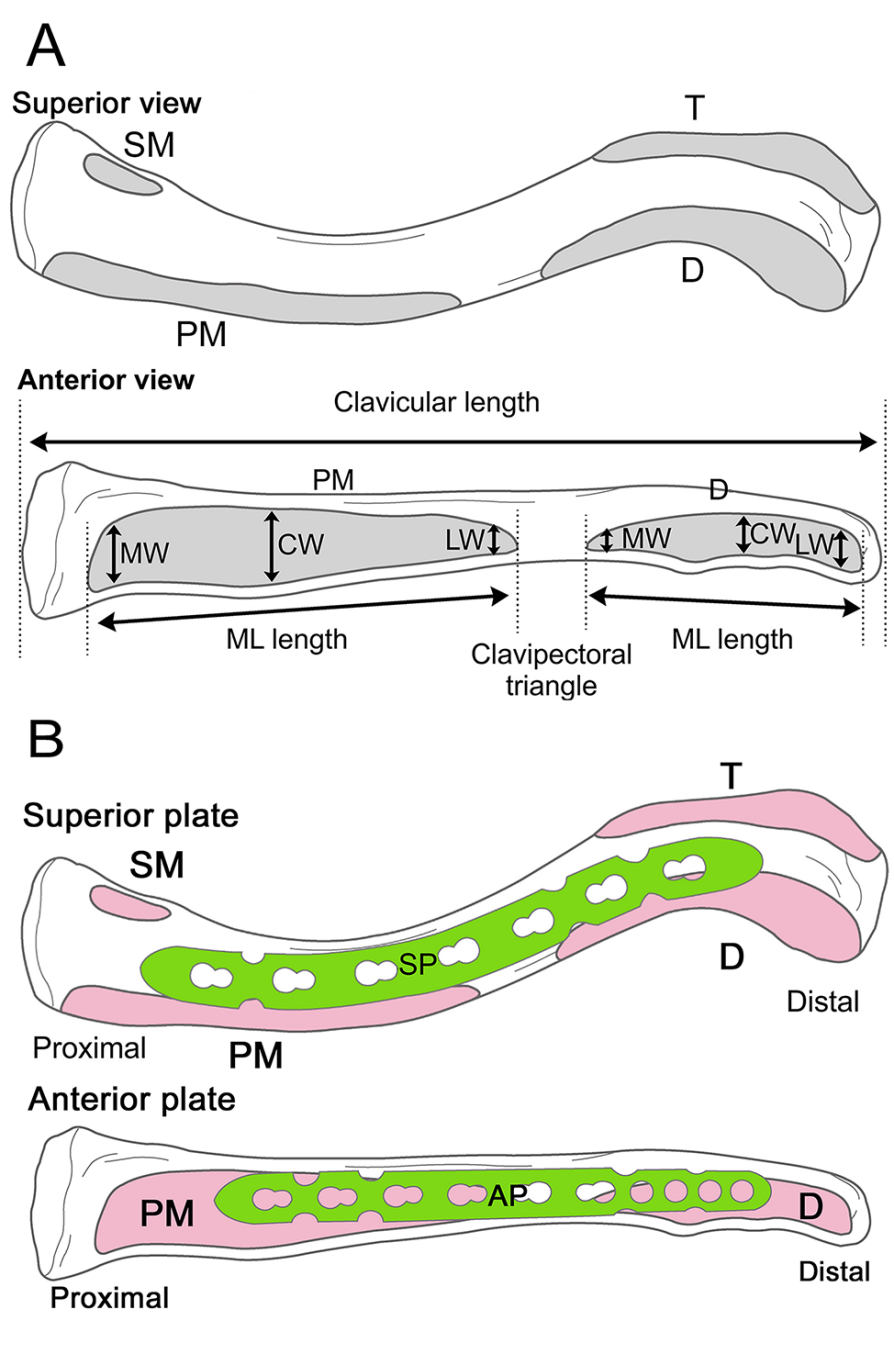
Scheme of the insertion of the muscles on the left clavicle (gray areas) A) Superior view. SM was attached to the proximal clavicle superiorly. The PM, T and D were partially attached to the superior clavicle. Most of the superior side of the clavicle show no attachment of these muscles. Representative schema of the measurement from the anterior view. Clavicular length, clavipectoral triangle length, and medial-to-lateral (ML) maximum length in D and PM muscles were measured. Anterior-to-posterior maximum medial width (MW), central width (CW), and lateral width (LW) in D and PM muscles were measured. These data were described in Table 1 B) Scheme of the templating of the superior plate (SP) (green) and insertion site of the muscles (pink) reconstructed by computed tomography (CT) in the left clavicle. Scheme of the templating of the anterior plate (AP) (green) and insertion site of the muscles (pink) reconstructed by CT in the left clavicle SM, sternocleidomastoid muscle; PM, pectoralis major muscle; D, deltoid muscle; T, trapezius muscle

We measured the clavicular length, maximum medial-to-lateral (ML) length, and maximum anterior-to-posterior (AP) width of the insertion part of the sternocleidomastoid, trapezius, pectoralis major, and deltoid muscles in 38 human clavicles using an electronic caliper (model: 19,977, Shinwa Sokutei, Niigata, Japan, resolution: 0.01 mm). The maximum AP width was measured at the medial (MW), central (CW), and lateral (LW) margins (Fig. 1A). The CW of the sternocleidomastoid muscle was not measured because of its small size. All measurements were performed in triplicate for each sample by three independent observers, and mean ± standard deviation values were calculated. Intraclass correlation coefficients (ICC) for each value were calculated to evaluate measurement accuracy within each observer.

**Table 1:** Measurement of the muscles surrounding the clavicle. The central width of the sternocleidomastoid muscle was not tested because it was too small. SD, standard deviation

Muscle	Sternocleidomastoid (mm) Mean ± SD	Trapezius (mm) Mean ± SD	Pectoralis major (mm) Mean ± SD	Deltoid (mm) Mean ± SD
Medial to lateral length	23.46 ± 7.94	52.58 ± 6.37	69.78 ± 15.54	46.37 ± 10.35
Medial width	8.98 ± 2.12	9.12 ± 2.16	9.18 ± 2.85	6.64 ± 1.96
Central width	Not tested	14.06 ± 3.25	11.43 ± 2.47	8.43 ± 3.12
Lateral width	6.15 ± 1.6	14.40 ± 5.61	7.58 ± 1.83	12.43 ± 3.80

### Reconstruction of the muscle insertion area on the clavicle and evaluation using 3D plate templating

All clavicles were scanned with IQon Spectral Computed Tomography (CT) (Philips Healthcare, Netherlands) at the University Hospital of Miyazaki (Miyazaki, Japan). A 3D model of the clavicles was reconstructed from CT data using the application software MIMICS 23.0 (Materialise, Leuven, Belgium). Using these data, the bone surface configuration concerning the insertion area of the muscles and the non-attachment area from the model were evaluated.

Generally, a medical device is used to fix clavicle fractures. We selected two types of plates: the anterior plate (VA-LCP Anterior Clavicle Plate, 10 holes, 101 mm, Synthes^®^, Tokyo, Japan) and the superior plate (LCP Superior Clavicle Plate, 7 holes, 110 mm, Synthes^®^, Tokyo, Japan), the lengths of which were sufficient to cover three or more holes in the proximal and distal parts. Three-dimensional templating was performed on both the superior and anterior clavicle plates using CT data (Fig. 1B). The area of coverage by the anterior and superior plates on the sternocleidomastoid, trapezius, pectoralis major, and deltoid muscles were measured using Materialise 3-matic 15.0 software (Materialise, Leuven, Belgium). The areas covered by these muscles were measured using the following formula: medial length × lateral length and were evaluated statistically.

### Histological analysis of the insertion sites of the muscles

We performed histological examination of the insertion sites of the sternocleidomastoid, trapezius, pectoralis major, and deltoid muscles in four randomly selected specimens. The specimens were immersed in Kalkitox (FUJIFILM Wako Pure Chemical Corporation, Osaka, Japan) for 8 h to undergo decalcification. Formalin-fixed and paraffin-embedded sections, 5 μm thick, were stained with hematoxylin and eosin.

### Statistical analysis

Comparisons between two unpaired groups were performed with Student’s t-test, using JMP Pro version 16 (SAS Institute Inc., Cary, NC). Statistical significance was set at *p* < 0.05. An ICC score above 0.75 was considered to indicate excellent agreement. All ICC score was evaluated as > 0.8 (range, 0.84–0.99).

## Results

### Measurement and evaluation of the insertion sites of the muscles attached to the clavicle

**Fig. 2:**
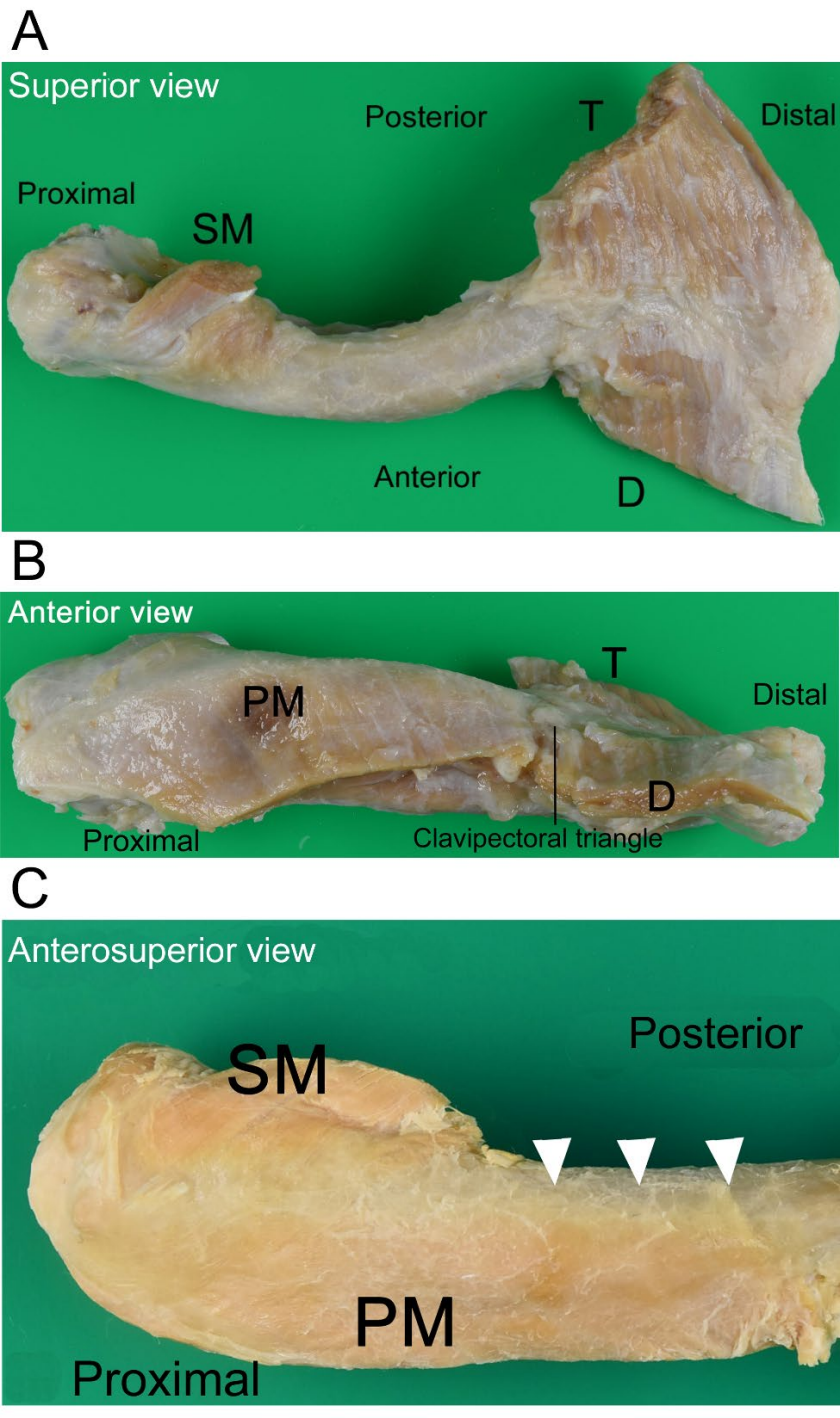
Left clavicle showing muscles dissected approximately 1 cm from the point of insertion A) Superior macrograph of the left clavicle T, trapezius; D, deltoid muscle; SM, sternocleidomastoid muscle. The border between T and D was unclear B) Anterior macrograph of the left clavicle PM, pectoralis major and T were attachedsuperiorly. It was difficult to distinguish the border between PM and D at the clavipectoral triangle. In some cases, the border between the PM and D was almost absent C) Anterosuperior macrograph of the proximal part of the left clavicle. The border between the clavicle and PM is unclear (white arrow)

The clavicular length was 151.6 ± 11.4 mm. The areas of all the insertion sites on the clavicle were evaluated as follows: the sternocleidomastoid muscle was attached proximally and superiorly (Fig. 2A); the trapezius muscle was attached posteriorly and partly superiorly (Fig. 2A); and the pectoralis major and deltoid muscles were attached anteriorly and partially superiorly (Fig. 2A and B). Next, we measured all muscle insertion areas attached to the clavicle, as described in Table 1. The mean values of the measurements were as follows: The ML length of the sternocleidomastoid muscle insertion was 23.46 ± 7.94 mm, MW length was 8.98 ± 2.12 mm, and LW length was 6.15 ± 1.6 mm. The ML length of the trapezius muscle insertion was 52.58 ± 6.37 mm, MW was 9.12 ± 2.16 mm, CW was 14.06 ± 3.25 mm, and LW was 14.40 ± 5.61 mm. The ML length of the pectoralis major muscles insertion was 69.78 ± 15.54 mm, MW was 9.18 ± 2.85 mm, CW was 11.43 ± 2.47 mm, and LW was 7.58 ± 1.83 mm. The mean ML length of insertion of the deltoid muscles was 46.37 ± 10.35 mm, mean MW was 6.64 ± 1.96 mm, CW was 8.43 ± 3.12 mm, and LW was 12.43 ± 3.80 mm. It was difficult to distinguish the attachment of the pectoralis major muscle and the border between the trapezius and deltoid muscles (Fig. 2A and C) macroscopically. The gap between the pectoralis major and deltoid muscles, called the clavipectoral triangle (Figs. 1A and 2B), was 29.60 ± 19.06 mm. All clavicles were highly individualized, with 5 out of the 38 cases not distinguishable in the clavipectoral triangle.

### Reconstruction of muscle insertion area of the clavicle and evaluation by 3D plate templating

**Fig. 3:**
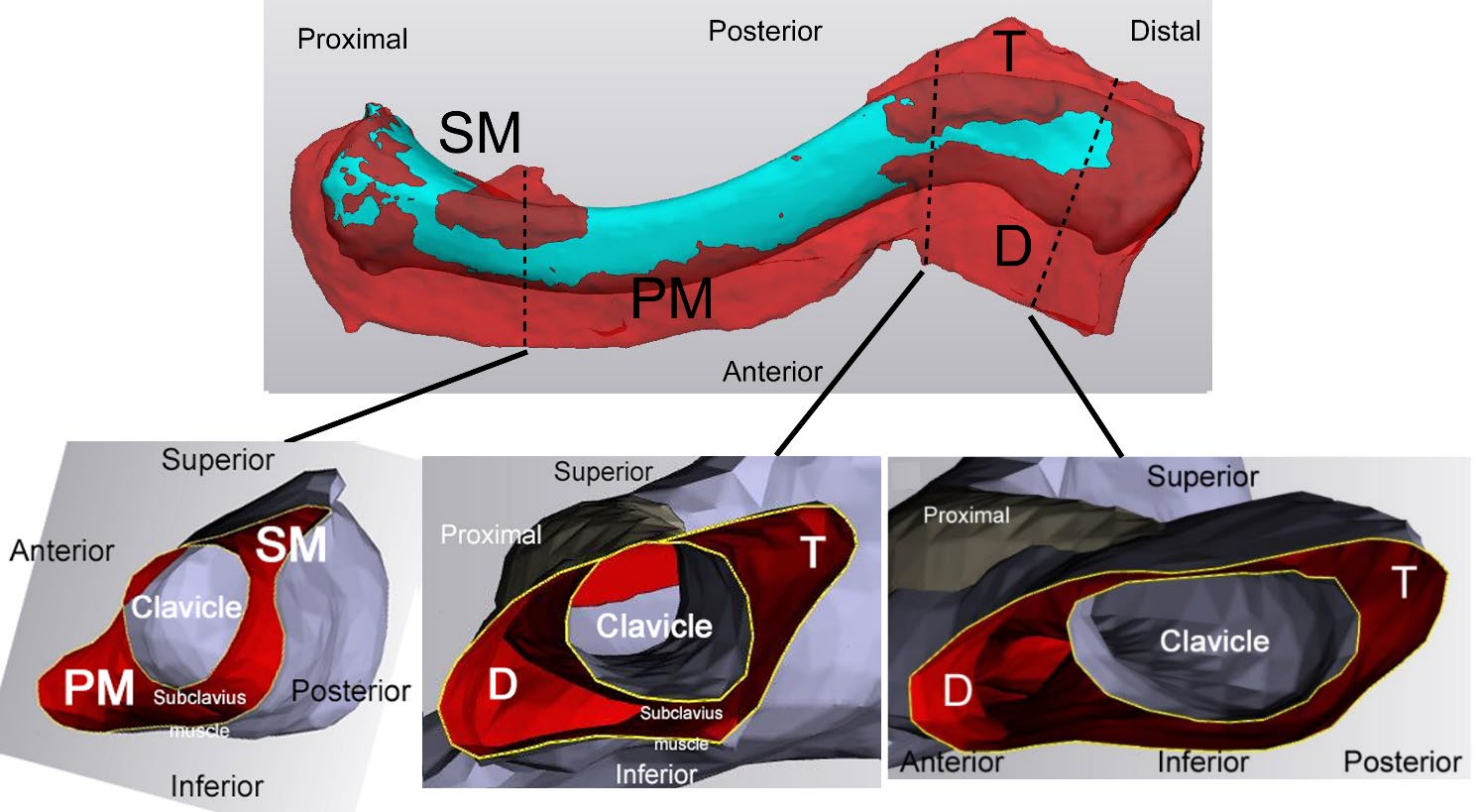
Superior view of left clavicle with clavicular muscle attachments reconstructed using MIMICS^®^. The dotted lines of the clavicle, show the sagittal views of the clavicle and surrounding muscles. SM, sternocleidomastoid muscle; PM, pectoralis major; T, trapezius; D, deltoid muscle

Sagittal 3D images of the clavicle model are shown in Fig. 3. In the proximal clavicle, the pectoralis major muscle was mainly inserted into the anterior area. In the middle to the distal clavicle, the deltoid was inserted anterosuperiorly, and the trapezius muscle was inserted posterosuperiorly. A non-attachment area was located in the middle of the posterosuperior clavicle. These results indicate that the insertions of the pectoralis major muscle and deltoid muscle were located at the front of the clavicle, whereas non-attachment areas were mainly identified in the superior area.

**Fig. 4:**
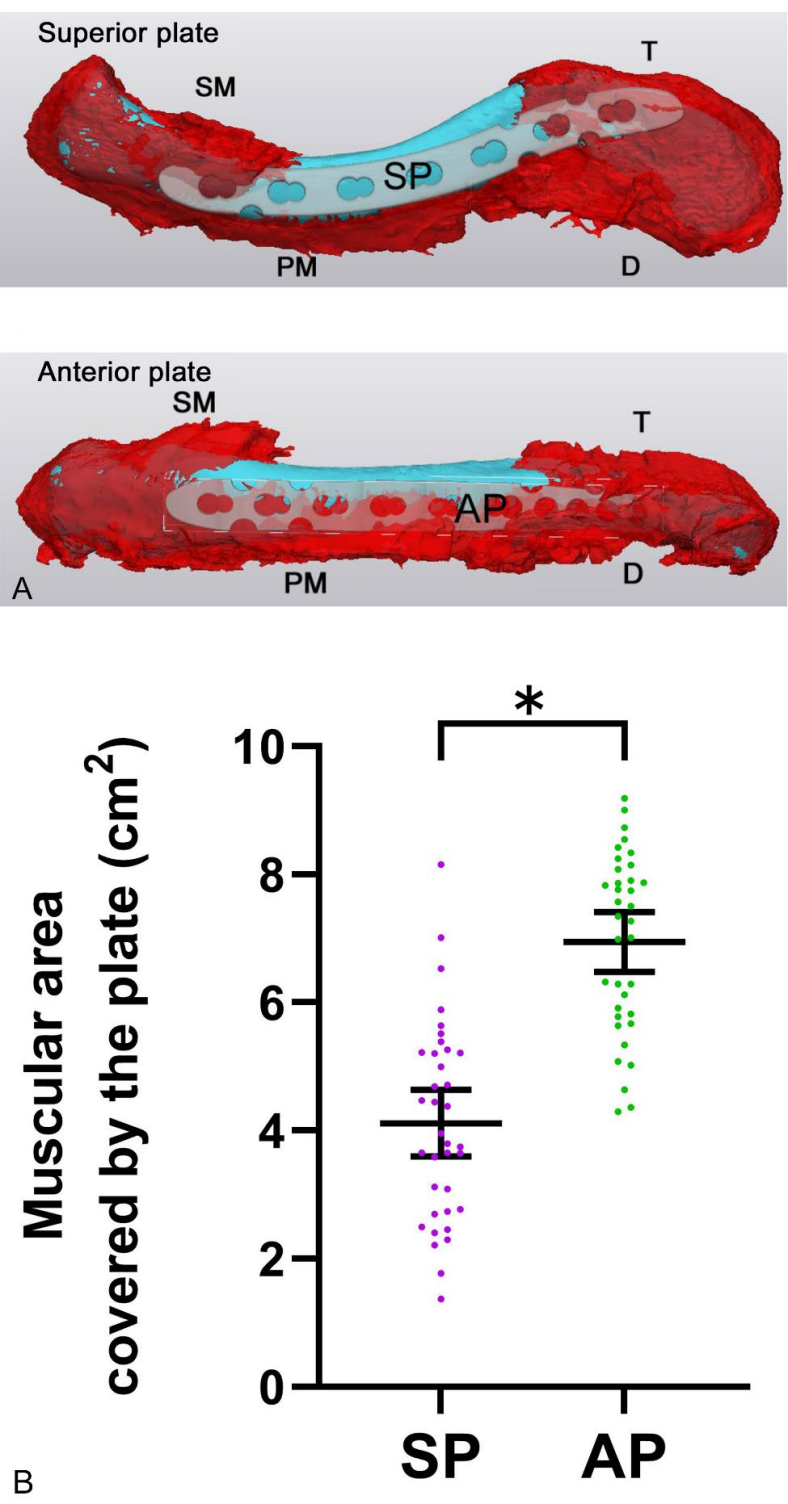
Images of the template of the plate (white area) on the clavicle (blue area) with muscular area (red area) reconstructed using MIMICS^®^. A) Images of the template of the superior and anterior plate B) Comparison of the covered area by the superior and anterior plate. The covered area by the superior plate was significantly smaller than that of the anterior plate. * p < 0.0001 (Student-t-test). Error bars indicate mean and 95% confidence interval SM, sternocleidomastoid muscle; PM, pectoralis major; T, trapezius; D, deltoid muscle; SP, superior plate; AP, anterior plate

Further, the superior and anterior plates were templated on the 38 clavicles in the 3D reconstruction (Fig. 4A). The mean area of the muscle attached to the clavicle covered by the superior plate was 4.11 ± 1.52 cm^2^, and that of the anterior plate was 6.94 ± 1.36 cm^2^. Statistically, the muscular area covered by the superior plate was significantly smaller than that covered by the anterior plate, with a mean difference of 2.827 (95% confidence interval, 2.138 to 3.516; p < 0.0001) (Fig. 4B). These data suggest that the anterior plate was prone to injure the pectoralis major and deltoid muscle attachments during the surgery.

### Histological analysis of the insertion site of the muscle

**Fig. 5:**
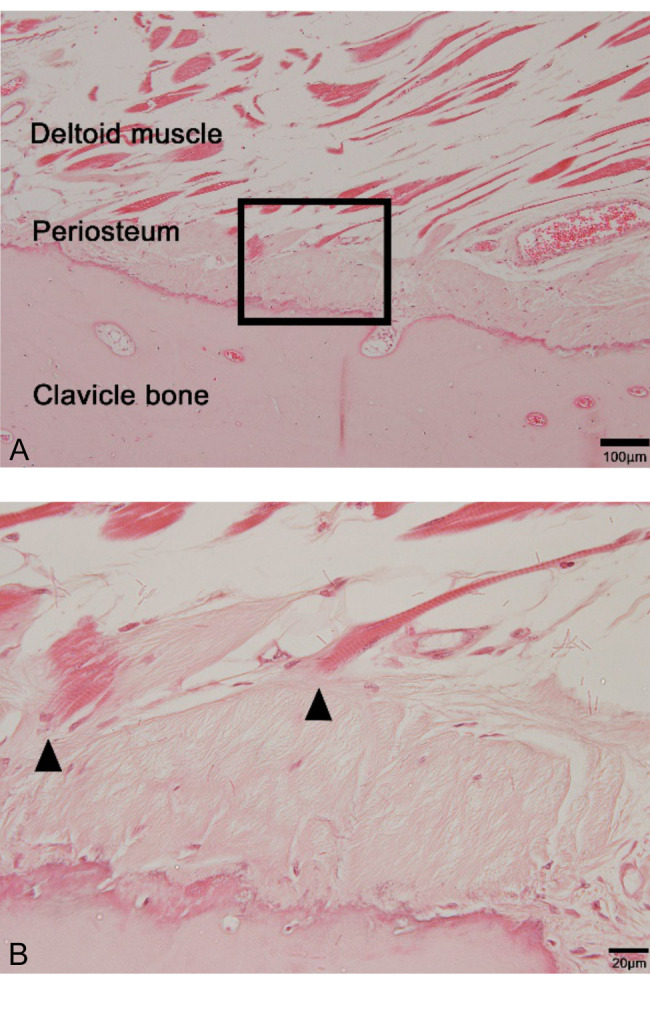
Micrographs of the clavicle stained with hematoxylin and eosin A) The border of the clavicle and insertion of the deltoid muscle showed fibrous enthesis. Scale bar = 100 μm. B) Magnification of the inset in figure A. The insertion of the deltoid muscle directly attached to the periosteum (arrows). Scale bar = 20 μm

All insertion sites were categorized as fibrous entheses, based on histology. Microscopically, these muscles were directly inserted into the periosteum (Fig. 5A), and the border between the periosteum and insertion was unclear (Fig. 5B). These findings may complement the significance of careful dissection of the muscle for clavicular fixation using a plate.

## Discussion

To the best of our knowledge, this is the first study to demonstrate a quantitative analysis of the area of muscular insertion on the clavicle. Traditionally, the treatment of clavicle shaft fractures is conservative; however, recent studies recommend operative treatment for displaced clavicle shaft fractures [2, 20, 25]. The Canadian Orthopaedic Trauma Society supports primary plate fixation of completely displaced clavicular shaft fractures in active adults [26]. Some authors have described the anterior approach to clavicle surgery [11], which carries the risk of vascular injury during drilling [6, 24] and supraclavicular nerve injury [12]. However, to the best of our knowledge, there is no description of preservation of muscle insertions. We measured the area of muscular insertion of the clavicle and performed a quantitative analysis of the muscular insertion area. Using these data, we reconstructed a 3D clavicle with the insertion area of the muscles illustrated to help in the planning of plate placement during surgery.

Well-known training programs such as bench presses [9] and push-ups [8] are often performed by young individuals, especially athletes, to train the pectoralis major and anterior fibers of the deltoid muscles. Furthermore, inclined bench press training has been shown to achieve greater electromyographic activation of the clavicular portion of the pectoralis major muscle [22]. The previous study also showed the muscle footprint and volume of the clavicle using CT images, with the attachment being variable [10], generally similar to our 3D images. Therefore, the findings of our study may guide surgeons who perform these procedures in terms of preserving muscle function, by applying our 3D analysis results to clinical treatment.

Some tools, such as intramedullary nails and superior and anterior plates, are used for　clavicle shaft fracture surgery. A recent biomechanical study suggested that plate fixation was stronger than nail fixation [13], whereas no significant differences in biomechanical results were observed between the superior and anterior plates [13]. Intramedullary nail is a suitable option for simple pattern fractures with better functional outcomes, lower infection rate, and no difference in union rate compared to plate fixation [7, 15, 17]. In contrast, when the clavicle fracture is either comminuted or long oblique, plate fixation is considered [7]. Our study clarified that the anterior plate covered a significantly broader area of muscle insertions attached to the clavicle than that of the superior plate. Accordingly, the superior plate may be a better choice for young individuals who want to preserve the function of the deltoid and pectoralis major muscles, as inclined bench press training mainly takes effect on the clavicular portion of the pectoralis major muscle [22]. A meta-analysis showed that using the anterior plate reduced blood loss, operation time, and union time compared with using the superior plate, whereas no differences were observed in the Constant score or rates of infection, nonunion, and complications [1]. The benefit of the anterior plate includes less skin irritation [13]. Further studies are needed to select an adequate fixation for the operative treatment of clavicle fractures.

We observed fibrous enthesis on macroscopic and microscopic images, suggesting that it was difficult to distinguish the border between the periosteum and the insertion of the muscles. Fibrous enthesis typically occurs over large surface areas in the diaphysis of long bones and is characterized by perforating mineralized collagen fibers [3]. Kumar et al. [16] showed that the deltoid muscle originates directly from its periosteum. A plate fixed over the surface of the clavicle inhibits blood supply, resulting in localized cortical bone necrosis [19]. Despite the further advancement of the limited contact dynamic compression plate, it also has adverse effects of pressure and friction on bone vascularity [23]. In other words, our histological findings indicated that a plate placed on or under the periosteum could invade the insertion. Our results suggest that the use of the superior plate, which covers smaller areas of muscle, may be more effective for blood supply and is suitable for muscle-sparing procedures.

This study had several limitations. First being the sample size as 38 cadavers are insufficient to represent all anatomical variations in bone and muscle. The cadaver donor was too old to require clavicle fracture surgery, and functional analysis after surgery was not performed, as we used only cadaveric data. However, the Constant score and Disability of the Arm, Shoulder and Hand scores used in previous studies [2, 26, 25] are insufficient for the functional analysis of the pectoralis major and deltoid muscles. The present results cannot account for intraoperative muscle injury because the muscle is dissected to expose and reduce fracture as well as the plate area. Our study is not only useful in the clinical setting of plate selection for clavicle shaft fractures, but also in the intramedullary nail fixation, other clavicle surgeries (such as for proximal and distal clavicle fracture, or acromioclavicular joint dislocation), and conservative treatment. Furthermore, it may have implications for conducting more basic science studies, such as in histology and anatomy. Further research is required to determine whether the anterior or superior plate is superior and to analyze function, focusing on muscle strength around the clavicle. It is important to consider that not all clavicle fractures require operative treatment [25] and that there is a risk of injury to the muscle insertion when either plate is chosen. Shared decision-making regarding the operative treatment options and selecting the appropriate tools for repairing clavicle fractures may be important considerations; however, the lifestyle of the patient should also play a role in determining the appropriate treatment.

## Conclusion

In conclusion, most of the pectoralis major and deltoid muscles were attached anteriorly. The sternocleidomastoid muscle was attached proximally and superiorly and the trapezius muscle was mainly attached posteriorly. The non-attachment area was located mainly in the posterosuperior part of the clavicle midshaft. Both macroscopically and microscopically, the boundaries between the periosteum and these muscles were difficult to demarcate. The anterior plate covered a significantly broader area of the muscles attached to the clavicle than that by the superior plate.

## Data Availability

All data are available from the corresponding author upon reasonable request.

## Abbreviations

3D: three-dimensional
ML: medial-to-lateral
AP: anterior-to-posterior
MW: (maximum) anterior-to-posterior width measured at the medial margin
CW: (maximum) anterior-to-posterior width measured at the central margin
LW: (maximum) anterior-to-posterior width measured at the lateral margin
ICC: intraclass correlation coefficients
